# Leaf surface microbiota transplantation confers resistance to coffee leaf rust in susceptible *Coffea arabica*

**DOI:** 10.1093/femsec/fiae049

**Published:** 2024-04-10

**Authors:** Leandro Pio de Sousa, Jorge Maurício Costa Mondego

**Affiliations:** Instituto Agronômico, Centro de Pesquisa e Desenvolvimento de Recursos Genéticos Vegetais, Campinas, 13020-902 São Paulo, Brazil; Instituto Agronômico, Centro de Pesquisa e Desenvolvimento de Recursos Genéticos Vegetais, Campinas, 13020-902 São Paulo, Brazil

**Keywords:** *Coffea*, coffee leaf rust, *Hemileia*, microbiota, phyllosphere, transplantation

## Abstract

Coffee leaf rust, caused by the fungus *Hemileia vastatrix*, has become a major concern for coffee-producing countries. Additionally, there has been an increase in the resistance of certain races of the fungus to fungicides and breeding cultivars, making producers use alternative control methods. In this work, we transplanted the leaf surface microbiota of rust-resistant coffee species (*Coffea racemosa* and *Coffea stenophylla*) to *Coffea arabica* and tested whether the new microbiota would be able to minimize the damage caused by *H. vastatrix*. It was seen that the transplant was successful in controlling rust, especially from *C. stenophylla*, but the protection depended on the concentration of the microbiota. Certain fungi, such as *Acrocalymma, Bipolaris, Didymella, Nigrospora, Setophaeosphaeria, Simplicillium, Stagonospora* and *Torula*, and bacteria, such as *Chryseobacterium, Sphingobium* and especially *Enterobacter*, had their populations increased and this may be related to the antagonism seen against *H. vastatrix*. Interestingly, the relative population of bacteria from genera *Pantoea, Methylobacterium* and *Sphingomonas* decreased after transplantation, suggesting a positive interaction between them and *H. vastatrix* development. Our findings may help to better understand the role of the microbiota in coffee leaf rust, as well as help to optimize the development of biocontrol agents.

## Teaser

Transplantation of leaf microbiota enhances *Coffea arabica* resistance against the coffee leaf rust fungus.

## Introduction

Coffee leaf rust (CLR) is caused by the multicellular basidiomycete fungus *Hemileia vastatrix*, an obligate parasite that affects the leaves of *Coffea arabica* (Avelino et al. 2006). The disease impairs photosynthesis, causing defoliation and weakening of the plant, which can reduce production by up to 80% (Avelino et al. 2006, Waller et al. 2007). CLR became a major concern for coffee-producing Latin American countries, especially after the great epidemic of 2012, which caused $1 billion in losses and affected >2 million people (Avelino et al. 2015).

Traditionally, the control of CLR has been carried out with the use of fungicides and tolerant cultivars of *C. arabica* (Zambolim 2016, de Resende et al. 2021). Nevertheless, there has been an increase in the resistance of certain races of the fungus to both fungicides and plant cultivars. Therefore, coffee producers are searching for alternative methods to control this disease (Sera et al. 2022). One of the proposed methods is the use of biological control agents, especially fungi and bacteria. CLR biocontrol can be achieved by the use of mycoparasites, especially the white halo fungus *Lecanicillium lecanii* (James et al. 2016), and coffee endophytic microorganisms that act directly as antagonists (Silva et al. 2012).

However, the use of these biocontrol agents faces certain difficulties, such as a low growth rate in artificial media (James et al. 2016) and a lack of reproducibility in the field of the results obtained under controlled conditions (Haddad et al. 2009). It is speculated that these unreproducible results are due to the low diversity of microorganisms used for biocontrol and the exclusion of the complex relationships within the microbe community that constitute the plant-microbe system (Armanhi et al. 2018). Therefore, it is a difficult task to choose the exact group of microbes that can provide protection to their hosts against pathogen attack.

Recently, our group showed that the diversity of fungi found on leaves of *Coffea racemosa* and *Coffea stenophyla*, which are resistant to *Phoma* spp. and *Hemileia vastatrix* (Melo et al. 2006), is much greater than that found on susceptible *Coffea arabica* (de Sousa et al. 2021). We hypothesized that there is a connection between fungal diversity and disease resistance. Therefore, the resistance to the pathogen is a combination between the composition of microbiota inhabiting the infection site and genetic factors intrinsic to the host. To evaluate this hypothesis, we transplanted the leaf surface microbiota of the CLR-resistant coffee species *C. stenophylla* and *C. racemosa* to *Coffea arabica* and tested whether it would be able to minimize the damage caused by *H. vastatrix*. In a second step, we detected which changes in the fungi and bacteria population occurred due to these transplants.

## Experimental procedures

### Preparation of biological materials

Mature young leaves of *Coffea arabica* cv. “Mundo Novo” (susceptible to all races of *Hemileia vastatrix*) were collected from adult plants in the active germplasm bank of the Instituto Agronômico de Campinas, Sao Paulo, Brazil (22^○^53'S 47^○^50'W, 664 m a.s.l.). Samples were collected on 14 April 2023, during autumn in the Southern hemisphere. Leaves were harvested from five adjacent plants, and were therefore submitted to the same climatic and soil (clayey oxisol) conditions. Leaf disks were cut with the aid of a cork borer, measuring 2.0 cm in diameter. Leaf disks were placed, with the abaxial surface facing upwards, in plastic boxes on a foam slide saturated with water. The box was closed with a glass slide. Uredospores of *H. vastatrix* were collected by scraping the surface of injured leaves of coffee plants, and then were homogenized in 0.9% NaCl to a concentration of 1 mg/ml. The uredospores were protected from light until inoculation.

### Collection and transfer of microbiota and *H. vastatrix* uredospores

For microbiota collection, leaves of *Coffea stenophylla* and *Coffea racemosa* were collected from adult plants in the active germplasm bank of the Instituto Agronômico de Campinas, Sao Paulo, Brazil (22^○^53'S 47^○^50'W, 664 m a.s.l.). Then 10 g of leaves was placed in a beaker containing 40 ml of saline solution (0.9% NaCl). The microorganisms were dislodged from leaves by sonication according to Morris et al. (1998), with modifications (70% strength for 6 min; pulsations 1 s off, 2 s on; 30 kHz). The resulting washes were concentrated by centrifugation at 10 000 g for 20 min at 4°C and the pellet was suspended in 500 µL of 0.9% NaCl. Two concentrations of washes were used: 100% of original wash and 10% of original wash. After the leaf disks were placed in the boxes, an aliquot of 25 µL of the suspensions was applied and then smoothly spread over the disks. After 24 h of incubation, 25 µL of uredospores (1 mg/ml) was applied and spread over the disks. The box was closed and left in the dark for 24 h to allow the uredospores to germinate. Thereafter, samples were incubated in a controlled environment with a photoperiod of 12 h, 500–1000 lux, 25 ± 2°C and relative humidity close to 100%. A completely randomized experimental design was used, with each treatment having three replicates represented by 10 leaf disks each. The data were analyzed using ANOVA, and differences among treatments were analyzed using the Tukey test (α = 0.05). The evaluation of disease symptoms was performed 30 days after inoculation.

### Uredospore germination inhibition tests

The *in vitro* experiment to evaluate whether phylosphere washes inhibit *H. vastatrix* uredospore germination inhibition consisted of five treatments: (i) wash without prior treatment; (ii) supernatant collected by centrifugation of the wash at 14 000 revs/min for 20 min; (iii) microbial cells collected (sediment after the centrifugation treatment described in (ii)) and resuspended in 0.85% NaCl saline solution; (iv) microbial cells collected from treatment (iii) and inactivated by exposure to ultraviolet radiation for 30 min; and (v) application of copper hydroxide (2.2 g/L). A 10-µL sample of each treatment + 10 µL of the uredospore suspension were placed in Petri dishes (5-cm diameter) containing 1.5% agar-water. The Petri dishes were maintained at 22°C in the dark. After 15 h, 5 µL of lactophenol was added to each dish to terminate the uredospore germination. The germination of 100 uredospores was observed using light microscopy. We considered a spore germinated when the length of the germination tube was equal or larger than the spore length. Each experiment was performed twice using a completely randomized design with three replications (one Petri dish = one experimental unit).

### Microbes collection, DNA extraction and sequencing

Thirty days after *H. vastatrix* infection, DNA samples from the surfaces of leaves were collected with sterilized cotton-tipped swabs moistened with 0.9% NaCl solution. DNA extraction was carried out with magnetic beads (MagMax® ThermoFisher Scientific, Waltham, MA, USA) according to the manufacturer's protocol. The identification of microbes was performed using high-performance sequencing of the fungal ITS1 region by amplification with ITS1 (GAACCWGCGGARGGATCA) and ITS2 (GCTGCGTTCTTCATCGATGC) primers (Schmidt et al. 2013) and V3-V4 of the bacterial 16S rRNA gene by amplification with 341F sequence (CCTACGGGRSGCAGCAG) and 806R sequence (GGACTACHVGGGTWTCTAAT). Libraries were prepared using TruSeq DNA Sample Prep Kits (Illumina, San Diego, CA, USA) and sequenced in a MiSeq system using the standard Illumina primers provided by the manufacturer. Paired-end 300 nucleotide runs were performed. All sequences were processed using QIIME2, according to Vernier et al. (2020). High-quality sequences (>200 bp in length, quality score >25 according to QIIME parameters) were trimmed and clustered into operational taxonomic units (OTUs) at 97% sequence identity using Mothur 1.48.0 (https://github.com/mothur/mothur/releases, accessed in June 2023). Representative sequences for each OTU were then aligned using PyNAST (Caporaso et al. 2010) and assigned taxonomy with UNITE and SILVA database to calculate strain abundances. Fungal and bacterial sequences are available in the NCBI Sequence Read Archive (www.ncbi.nlm.nih.gov/sra) under the accession number PRJNA1004833.

### Diversity metrics, ordination, clustering, classification and statistical analysis

The pre-processing, summarization, normalization, diversity metric, ordination/clustering, sample classification and significance testing were carried out using the web-based MicrobiomeAnalyst pipeline (Dhariwal et al. 2017). Alpha diversity (measured within sample diversity) was computed using OTU richness and Shannon diversity. To detect differences in richness between groups, we used the Kruskal–Wallis rank sum test, and the filtered data were converted into relative abundance. Beta diversity (a measure of microbial compositional differences between samples) was computed using the Bray–Curtis dissimilarity metric. Permutational Analysis of Variance (PERMANOVA) was used to access significant differences between treatments and the control. The composition (presence/absence) and abundance of each OTU analysis between the transplant-treated samples and the non-transplant-treated control were performed based on nonmetric multidimensional scaling (NMDS) with the Bray–Curtis distance metric (beta diversity) using the VEGAN package in R software (Dixon 2003). ADONIS, vegan package in R, was used with 999 permutations to quantify the effect size of variables explaining Bray–Curtis distance. The DESeq2 package was used to evaluate the univariate differential abundance of OTUs (Love et al 2014). An OTU was considered differentially abundant if adjusted *P* values were <0.05 and the absolute value of the logFC ≥1. We only used OTUs with relative abundance >1% to prevent infrequent genera from distorting the final results. Microbial co-occurrence networks were generated using the Cytoscape program (Faust and Raes 2016) based on pair-wise correlations between abundances of different microbial genera. The Spearman correlation coefficient (ρ) was used to obtain the pair-wise correlations between relative abundances of the microbial genera.

## Results

**Figure 1.**
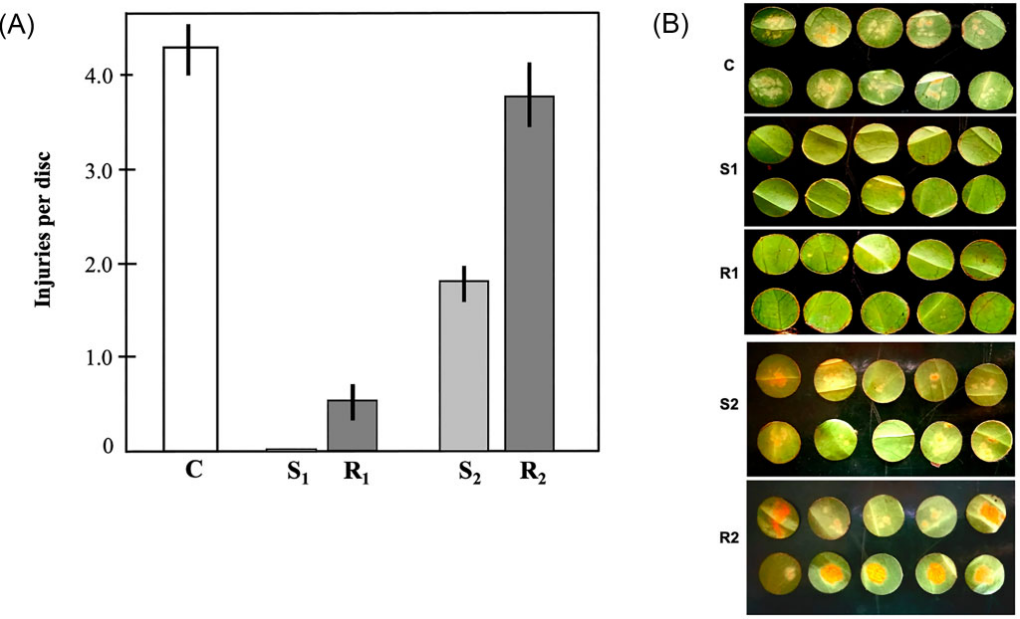
Evaluation of coffee leaf rust (CLR) symptoms after leaf surface microbiota transplant. (A) Severity of CLR in transplanted and non-transplanted leaf disks evaluated by the average number of injuries per disk 30 days after inoculation. Vertical bars symbolize standard deviations (Tukey α = 0.05). (B) Visual analysis of transplanted and non-transplanted leaf disks. C = Control; S1 = transplantation of *C. stenophylla* leaf surface without dilution; R1 = transplantation of *C. racemosa* leaf surface without dilution; S2 = transplantation of *C. stenophylla* leaf surface with washing dilution in 1 : 10; R2 = transplantation of *C. racemosa* leaf surface with washing dilution in 1 : 10.

We conducted *in vitro* experiments to verify whether transplantation of microbiota from wild and CLR-resistant coffee plants would confer some degree of protection to *C. arabica*. We verified that leaf surface microbiota transplantation of *C. racemosa* (R1) and *C. stenophylla* (S1) conferred protection to *C. arabica* against CLR (Fig. 1A). Leaf disks transplanted with S1 showed the lowest number of lesions. *Hemileia vastatrix* caused little or no lesions at the time of the experiment (Fig. 1A and B). Preliminary data suggest that this phenomenon also occurs in plantlets (data not shown). Therefore, we suggest that microbiota transplantation may confer protection against CLR by increasing fungal diversity on leaves, at least in experiments conducted in a controlled environment. We also verified that the antagonistic effect of transplantation depends not only on the donor, but also on the cell concentration (Fig. 1A and B). As seen in Fig. 1, when transplanting with 1/10 of the initial concentration (R2 and S2), the protective effect decreases and, in the case of *C. racemosa* (R2) transplantation, the protective effect was almost lost (Fig. 1A and B). Finally, we verified that the phyllosphere wash (either with or without cells) did not decrease the germination of *H. vastrix* spores (Table 1), suggesting another mechanism for the antagonism.

**Table 1.** Effect of phyllospheric wash on uredospore germination. Spore germination was evaluated by placing spores in the presence of (i) wash without prior treatment; (ii) supernatant collected by centrifugation of the wash at 14 000 revs/min for 20 min; (iii) microbial cells collected (sediment after the centrifugation treatment described in (ii)) and resuspended in 0.85% NaCl saline solution; (iv) microbial cells collected from treatment (iii) and inactivated by exposure to ultraviolet radiation for 30 min; and (v) application of copper hydroxide (2.2 g/L). ^a^(Tukey's test, α = 0.05).

Treatment	Germination (%)		
	*C. racemosa*	*C. stenophylla*	*C. arabica*
Wash (i)	97.5 ± 0.3^a^	98.3 ± 0.2^a^	98.5 ± 0.3^a^
Supernatant (ii)	97.8 ± 0.1^a^	96.4 ± 0.2^a^	98.8 ± 0.2^a^
Microbial cells (iii)	98.1 ± 0.2^a^	97.2 ± 0.5^a^	97.4 ± 0.4^a^
Microbial cells inactivated (iv)	98.2 ± 0.3^a^	98.5 ± 0.1^a^	96.9 ± 0.5^a^
Copper hydroxide (v)	7.0 ± 1.1	6.7 ± 0.3	8.4 ± 1.3

Obs: Saline Treatment= 98.3 ± 0.2% of spores germinated

In addition to verifying the effect of the microbiome on CLR infection, we also verified the microbial structure differences between treatments using 16S and ITS sequencing. After discarding chimera sequences and sequences with barcode mismatches >1.5 or N bases, in total we sequenced 72 270 16S rDNAs, of which 390 were not classified and therefore were discarded, and 42 188 ITSs, of which 376 were not classified and also discarded.

**Figure 2.**
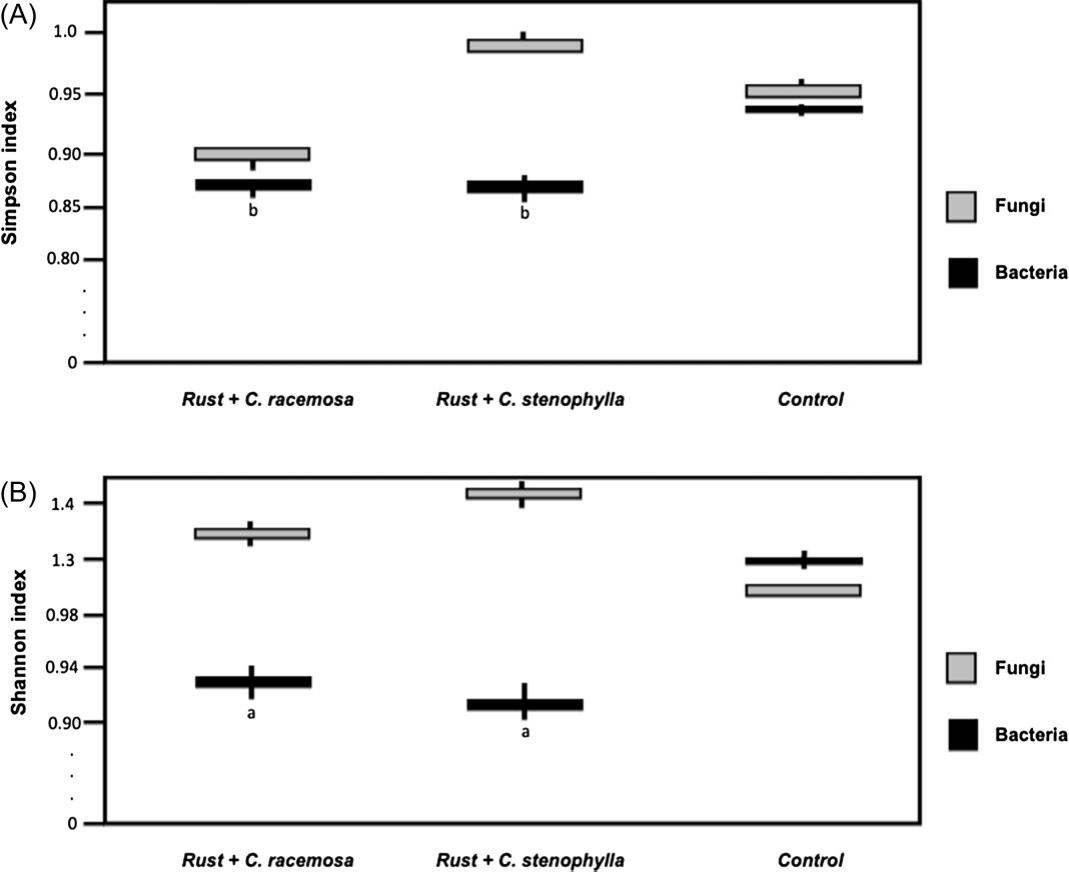
Alpha diversity for bacteria and fungi using the Simpson and Shannon index. “Control” means that there was only the inoculation of the pathogen. (A) Simpson index; (B) Shannon index. Significance for ANOVA in groups: a, b→ *P* < 0.01.

**Figure 3.**
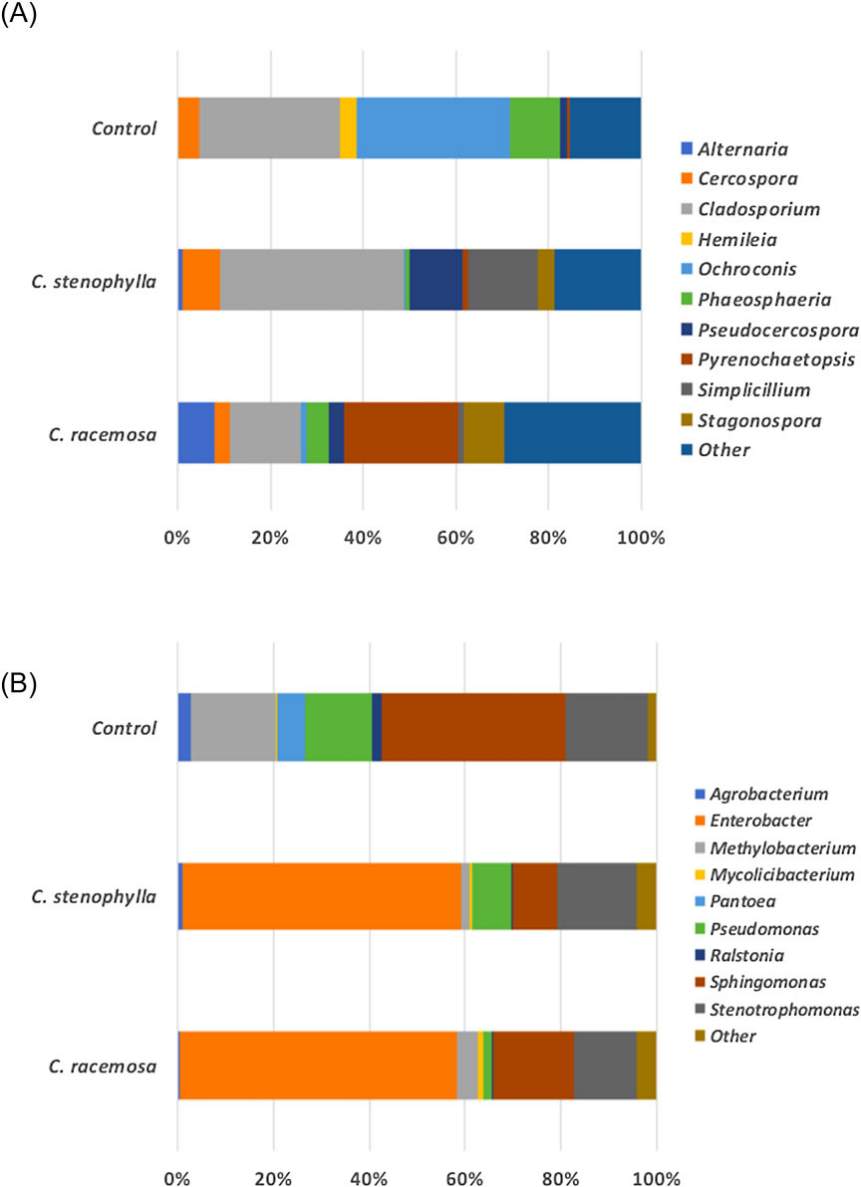
Relative abundance and microbiota distribution before and after leaf surface microbiota transplant. (A) Fungal and (B) bacterial relative abundance/distribution in *C. arabica* disks that received leaf surface microbiota transplants.

**Figure 4.**
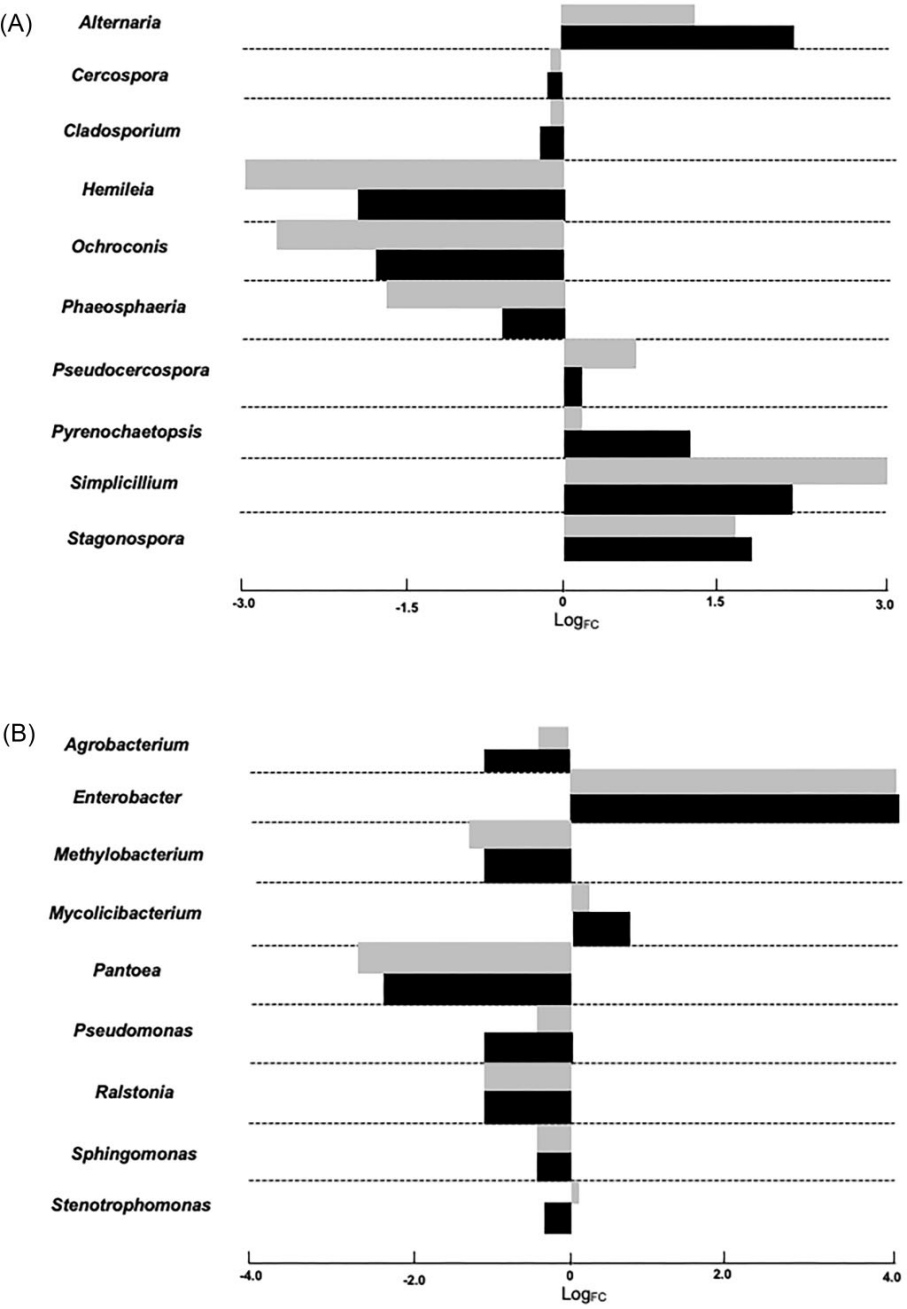
Quantitative analysis of microbial relative abundances after leaf surface microbiota transplants. Changes in (A) bacteria and (B) fungi populations. The estimated degree of differential abundance is represented by Log2 fold change (logFC), which indicates a positive or negative interaction (logFC > 0 or <0) of the specified OTU (each bar represents an OTU belonging to each genus) when the phyllosphere transplant was performed. The change is seen by comparing the transplant with the control. The gray and black bars represent the changes caused by *C. stenophylla* and *C. racemosa* transplants, respectively; *P* < 0.05.

**Figure 5.**
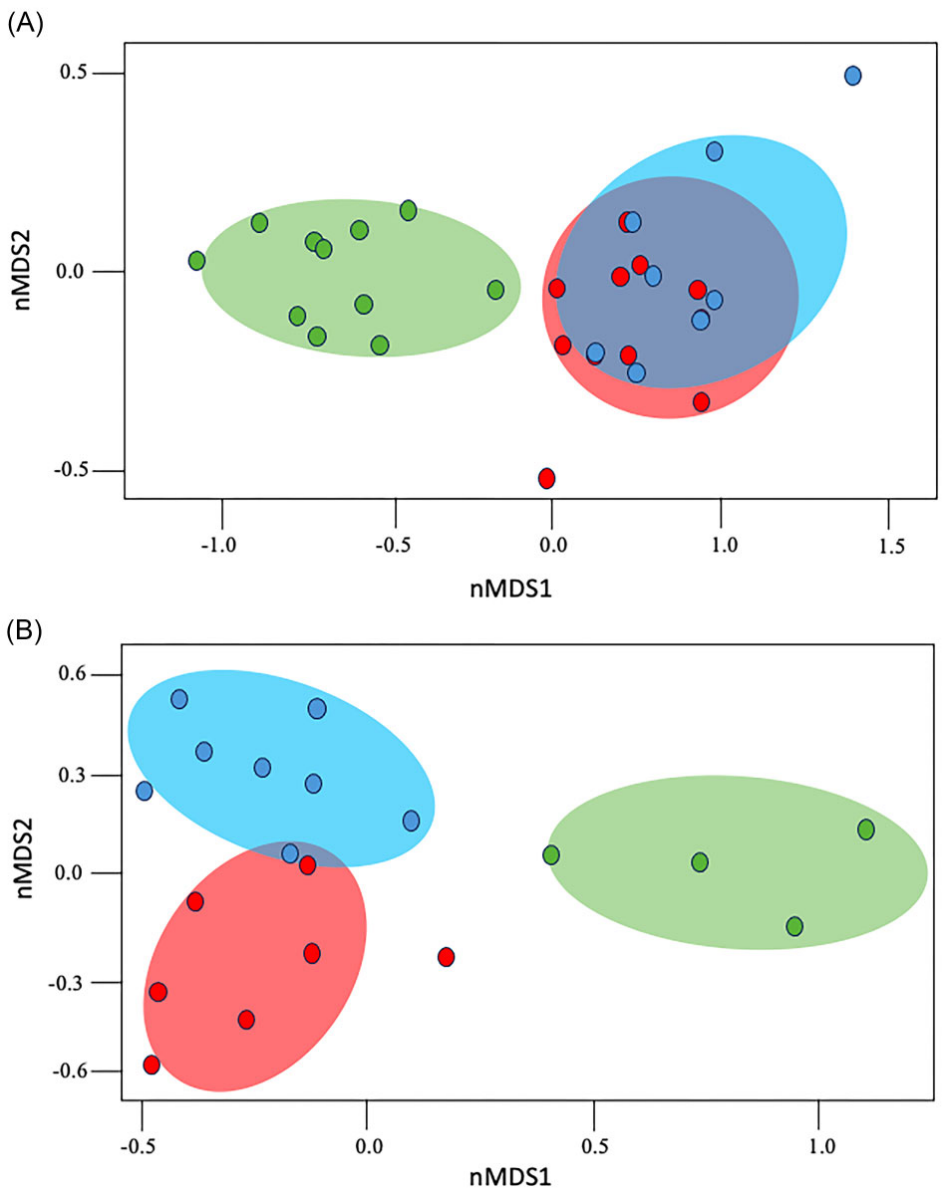
Beta diversity analysis of microbiota after leaf surface transplant. NMDS plots were constructed based on Bray–Curtis dissimilarity of (A) fungi and (B) bacteria OTUs. Control “no transplant” (green); *C. stenophylla* transplant (blue); *C. racemosa* transplant (red). The fungi community appeared to vary significantly between treatments (PERMANOVA, *P*  < 0.01). The bacteria communities from *C. stenophylla* and *C. racemosa* transplantations did not vary significantly from each other (PERMANOVA, *P* > 0.01).

In the case of the mycobiome (Fig. 2), the diversity in transplanted disks increased in relation to the control disk (with the exception of the Simpson index for the transplanted phyllosphere of *C. racemosa*), which corroborates our initial hypothesis that the greater diversity of fungi in wild coffee trees may contribute to resistance against CLR. The genera *Alternaria, Simplicillium* and *Stagonospora* had large increases in abundance (LogFC > 1.5) in disks with transplants from *C. racemosa* and *C. stenophylla* (Figs 3A and 4A). On the other hand, the genera *Ochroconis, Phaeosphaeria* and *Hemileia* showed a decrease in relative abundance (LogFC < –1.5; Fig. 4B). In addition, the mycobiome beta diversity analysis indicates significant differences between *C. racesoma* and *C. stenophylla* leaf surface transplants (Fig. 5A).

In the case of bacteriome, the scenario changes. The diversity of bacteria declined with the application of the leaf surface transplants (Fig. 3B). Transplantation with *C. racemosa* and *C. stenophylla* leaf phyllospheres led to similar changes and a decline in diversity (Fig. 3B). For example, in both transplants, *Enterobacter, Sphingomonas* and *Stenotrophomonas* were the prevalent genera, while in the control, *Methylobacterium* and *Pseudomonas* replaced *Enterobacter* among the prevalent genera. Additionally, the application of exogenous leaf surface microbiota led to an increase in certain bacterial groups, especially *Enterobacter* (logFC > 4; Fig. 4B), while other genera decreased, for example, *Methylobacterium, Ralstonia* and expressly *Pantoea* (logFC < –2.0; Fig. 4B). The bacteriome similarities between the two transplants are reinforced by the beta diversity analysis (Fig. 5B).

**Figure 6.**
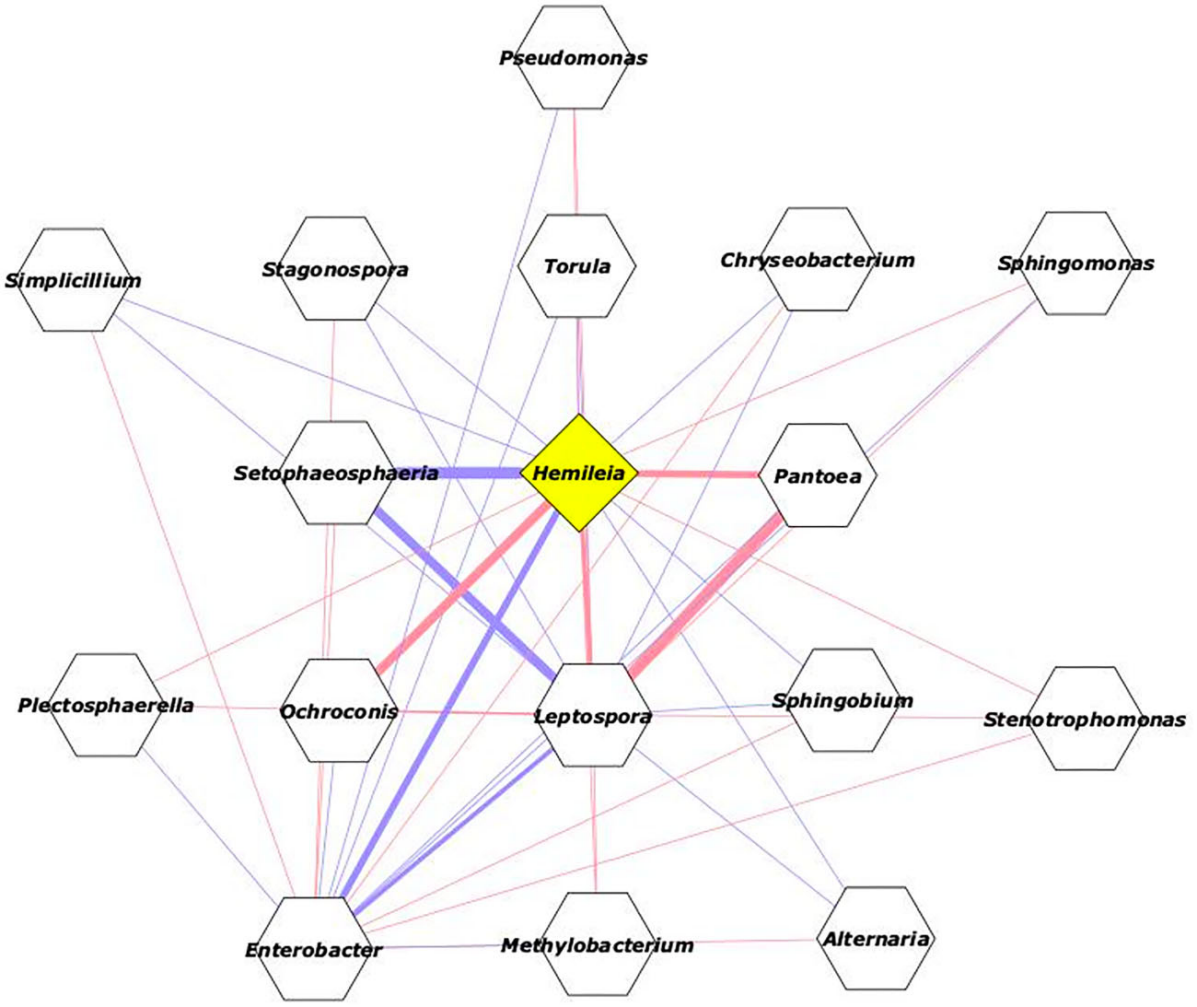
Cytoscape network analysis of transplanted microbiota. In blue: negative interactions; in red: positive interactions. The thickness of the line represents the intensity of the interaction.

Genera with relative abundance >1.0% were used to check for correlation networks derived from our experiment (Fig. 6). This previous filtering step removed under-represented OTUs and reduced network complexity, facilitating the visualization of the inter-relationship between the most relevant microbial groups. The network analysis showed that *Hemileia* forms a correlated group with *Ochroconis, Pantoea* and *Leptopora*, where all of them decline in population with the application of the transplant. On the other hand, *Enterobacter* and *Setopaeosphaeria* showed the greatest negative interaction with *Hemileia* (Fig. 6).

## Discussion

In our previous work, we hypothesized that the different responses of coffee plants to infection by *Phoma* could be related to the diversity of fungi in the phyllosphere (de Sousa et al. 2021). Thus, the susceptibility to this disease in *C. arabica* could be partially explained by the lower diversity of fungi in the phyllosphere, differing from resistant *C. racemosa* and *C. stenophylla*, which have greater diversity. Besides being resistant to *Phoma* spp., *C. racemosa* and *C. stenophyla* are also resistant to *H. vastatrix* (Melo et al. 2006). To evaluate this hypothesis, we transferred leaf microbiota from *C. racemosa* and *C. stenophylla* to *C. arabica*, inoculated *H. vastatrix*, measured the level of infection and evaluated the fungal and bacterial OTU changes. Despite the original premise involving only fungi, we decided to also analyze the change in the bacterial community, because mycobiome and bacteriome occupy the same physical spaces in the leaves (Lindow and Brandl 2003).

The transplant was successful in reducing CLR symptoms, especially when the donor was *C. stenophylla*, and it was dependent on the microbiota concentration. In fact, protection against pathogens depends on the high concentration of the biocontrol agent, otherwise the desired effect is not achieved (Bressan and Figueiredo 2008). Some fungi detected in our work are alleged as biocontrol agents and growth promoters, for example, *Acrocalymma* (Jin et al. 2018) and *Simplicillium* (Ward et al. 2012, Le Dang et al. 2014), which was isolated as an endophytic fungi of coffee trees (Gomes et al. 2018). However, the majority of fungi detected herein are important pathogens of other plants, such as *Alternaria* (Thomma 2003), *Bipolaris* (Bhunjun et al. 2020), *Didymella* (Keinath 2011), *Nigrospora* (Wang et al. 2017) and *Stagonospora* (Solomon et al. 2006). This finding is not completely surprising because several pathogenic fungi can also act as biocontrol agents depending on the strain and the host (Sajeena et al. 2020). For instance, *Alternaria* is predominantly saprophytic and is commonly found in the phyllosphere of healthy plants, making it part of the normal mycobiota (Uddin and Chakraverty 1996, Thomma 2003).

Despite the alterations that transplantation caused in the mycobiome, some groups had few abundance changes, especially *Cladosporium*, suggesting that the addition of an exogenous microbe (either by inoculation of a pathogen or by microbiota transplantation) does not significantly alter the population of this fungus. Indeed, Hladnik et al. (2023) found *Cladosporium* in large numbers on both infected and symptomless olive leaves. On the other hand, transplantation led to a large decrease in the populations of several genera, especially *Ochroconis* and *Hemileia*, as expected. Thus, it is reasonable to assume that the members of this group of fungi are, in some way, connected to each other (Fig. 6).

While transplantation led to an increase in fungal diversity, the bacteriome diversity decreased (Fig. 1). We verified that phyllospheric-related bacteria (Li et al. 2022), *Methylobacterium, Sphingomonas Pantoea* and *Pseudomonas*, prevailed in infected disks. However, after leaf surface transplantation, *Methylobacterium, Sphingomonas* and, especially, *Pantoea* populations were reduced, concomitantly with *H. vastatrix* symptoms (Figs 3 and 4). Therefore, we can hypothesize that these bacteria may be linked to the CLR. On the other hand, the *Enterobacter* population increased after transplant, suggesting that the bacteria of these genera may have a suppressive effect on CLR (Fig. 4). It is interesting to note that Hunter et al. (2010) found that, in the leaves of different lettuce varieties, *Enterobacter* and *Pantoea* had a negative correlation, while *Pseudomonas* showed little variation, with the same occurring in the present work. They also found that the *Erwinia* population (an important foliar pathogen) decreased when the *Enterobacter* population was high, suggesting perhaps that *Enterobacter* has some role in controlling pathogens in general.

The mechanism that can explain this antagonism remains unknown; however, the experiment with the germination of *H. vastatrix* spores may be a clue (Table 1). We verified that the presence of microbial cells and their metabolites alone do not act directly on the germination of spores, at least not in *in vitro* experiments. Given this, we offer two hypotheses for the antagonism seen in leaf disks: (i) inhibition of germination can occur *in vivo*, but not *in vitro* (Shiomi et al. 2006, Haddad et al. 2013). In this case, the establishment of the microbiota in the leaf may be necessary for the inhibition of spore germination to occur, so that we would have competition between the microbiota and *H. vastrix* for the best niches, especially close to the stomata; and (ii) indirect effect of the microbiota, where the alteration of the population of a certain non-target organism leads to alteration of the pathogen population (Ao et al. 2022, Dong et al. 2022), because we actually saw a strong correlation between *H. vastrix* and the group composed by *Pantoea* and *Ochroconis* (Fig. 6). However, we do not know if the decrease of *H. vastrix* causes the decrease of the other group members or vice versa. This is an interesting point to be investigated in the future because if the decline in the populations of *Pantoea* and *Ochroconis* is related to the decrease in the *H. vastrix* population then these other microbes can be targets in the search for more effective control of CLR.

Therefore, changes in the structure of the microbial community, both qualitatively and quantitatively, can influence the development of CLR.

The present work suggests that the transplantation of leaf microbiota from rust-resistant coffee trees to CLR-susceptible *C. arabica* also transfers some protection against the pathogen. We also show the dynamics of microbial populations and their inter-relationships, which are quite complex and not studied in the field of CLR control. Our findings provide a better understanding of the role of the microbiota in CLR and can help with the optimization of synthetical consortia used in biological control.
